# Clinical efficacy of N-acetylcysteine for COVID-19: A systematic review and meta-analysis of randomized controlled trials

**DOI:** 10.1016/j.heliyon.2024.e25179

**Published:** 2024-01-26

**Authors:** Ting-Hui Liu, Jheng-Yan Wu, Po-Yu Huang, Ya-Wen Tsai, Wan-Hsuan Hsu, Min-Hsiang Chuang, Hung-Jen Tang, Chih-Cheng Lai

**Affiliations:** a Department of Psychiatry, Chi Mei Medical Center, Tainan City, Taiwan; b Department of Nutrition, Chi Mei Medical Center, Tainan City, Taiwan; c Department of Internal Medicine, Chi Mei Medical Center, Tainan City, Taiwan; d Center for Integrative Medicine, Chi Mei Medical Center, Tainan, Taiwan; e Division of Hospital Medicine, Department of Internal Medicine, Chi Mei Medical Center, Tainan, Taiwan; f School of Medicine, College of Medicine, National Sun Yat-sen University, Kaohsiung, Taiwan

**Keywords:** COVID-19, N-acetylcysteine, SARS-CoV-2, Randomized controlled trial

## Abstract

**Background:**

The association between N-acetylcysteine (NAC) and COVID-19 remains undetermined; therefore, this meta-analysis assessed the clinical efficacy of NAC in the treatment of patients with COVID-19.

**Methods:**

This study searched PubMed, Embase, the Cochrane Library, and ClinicalTrials.gov for studies published from their inception to December 17, 2022. Only randomized controlled trials (RCTs) that assessed the clinical efficacy of NAC for patients with COVID-19 were included.

**Results:**

Five RCTs involving 651 patients were included. There was no significant difference in mortality between the study group receiving NAC and the control group (15.6 % [50/320] vs. 32.3 %, [107/331]; risk ratio [RR]: 0.58; 95 % confidence interval [CI]: 0.24–1.40). In addition, the two groups did not differ with respect to the incidence of invasive mechanical ventilation (RR: 0.93; 95 % CI: 0.65–1.33), the risk of intensive care unit (ICU) admission (RR: 0.86; 95 % CI: 0.62–1.21), the length of hospital stay (mean difference [MD]: 0.17 days; 95 % CI: −0.67–1.01), and the length of ICU stay (MD: −0.77 days; 95 % CI: −2.97–1.42).

**Conclusions:**

The administration of NAC did not improve the clinical outcomes of patients with COVID-19; its routine use is not recommended for patients with SARS-CoV-2 infections.

## 1 Introduction

More than 770 million confirmed cases of COVID-19 have been reported globally in the end of 2023 [[1], [2], [3]]. The World Health Organization has reported there have been over 6 million COVID-19-related deaths [1]. Researchers have developed several novel antiviral agents, namely remdesivir, molnupiravir, and nirmatrelvir plus ritonavir, for the treatment of non-hospitalized patients with COVID-19. In addition, systemic corticosteroids and interleukin-6 blockade are recommended for hospitalized patients with severe disease to mitigate the inflammatory and oxidative responses [4,5]. However, effective treatments against COVID-19 remain limited [6,7], and some anti-SARS-CoV-2 agents are expensive. Therefore, studies have assessed the treatment potential of several safe, affordable, and easily available drugs, such as colchicine, metformin, ivermectin, and fluvoxamine, for patients with COVID-19 [8,9].

N-acetylcysteine (NAC), a precursor in the synthesis of reduced glutathione, possesses mucolytic properties and exhibits a broad range of antioxidant and anti-inflammatory activities [10,11]. NAC has been recommended in the treatment of numerous diseases without safety concern. Therefore, increasing scientific interest in NAC has prompted the evaluation of its efficacy in COVID-19 studies [11,12]. An in vitro study demonstrated that NAC can reduce the inflammasome activation induced by SARS-CoV-2 [13], and several cohort studies have indicated that NAC therapy helped improve clinical recovery and oxygenation parameters, reduced the levels of inflammatory markers such as C-reactive protein and ferritin, and decreased the length of hospitalization [[14], [15], [16]]. Although additional randomized controlled trials (RCTs) have investigated the clinical efficacy of NAC in the treatment of patients with COVID-19, their findings were not consistent [[17], [18], [19], [20], [21]]. An RCT by de Alencar et al. reported that the NAC administration did not reduce the need for invasive mechanical ventilation (IMV), the rate of admissions to the intensive care unit (ICU), or the mortality rate in patients with severe COVID-19 [17]. In contrast to those of de Alencar et al., the results of Panahi et al. indicated that NAC helped decrease the mortality rate and inflammatory parameters level and reduced the risk of severe respiratory failure in patients with COVID-19 [20]. This systematic review and meta-analysis of RCTs addresses this controversy and provides robust evidence regarding the clinical efficacy of NAC for patients with COVID-19.

## 2 Methods

This study was conducted in accordance with the Preferred Reporting Items for Systematic Reviews and Meta-Analyses 2020 statement [22] and was registered in PROSPERO, an international database of prospectively registered systematic reviews (CRD42023388482).

### 2.1 Search strategy

This study searched PubMed, Embase, the Cochrane Library, and ClinicalTrials.gov for studies published from their inception to December 17, 2022. We also manually searched for additional eligible studies from the reference lists of relevant articles. The following medical subject headings were employed: “coronavirus disease 2019,” “COVID-19,” “SARS coronavirus 2 infection,” “SARS-CoV-2 disease,” “nCoV 2019 infection,” “2019-nCoV disease,” “acetylcysteine,” “fluimucil,” and “n-acetyl-l- cysteine” (Appendix 1).

Two researchers (THL and JYW) independently screened the titles and abstracts of the articles identified using the aforementioned search strategies to assess the potentially eligible studies. Disagreements were resolved by a third investigator (CCL). The researchers then obtained and reviewed full-text copies of potentially relevant articles for eligibility. Full-text copies of potentially relevant articles were then obtained and reviewed for eligibility. Studies were not excluded based on language, age, sex, race, or ethnicity.

### 2.2 Eligibility criteria

This meta-analysis included RCTs comparing NAC with a placebo or standard of care (SOC) in the treatment of patients with COVID-19. Included studies met the following criteria: (i) included patients with COVID-19; (ii) used NAC as an intervention; (iii) used a placebo or the SOC for comparison; and (iv) reported clinical efficacy as the study outcomes. This meta-analysis excluded the following: (i) conference posters, case reports, case series, and observational studies; (ii) single-arm studies; (iii) studies that did not report the outcomes of interest; (iv) pharmacokinetic studies; and (v) studies in which the comparison group did not receive a combination treatment similar to NAC in the intervention group.

### 2.3 Data extraction

Researchers extracted the following information from the articles: the study design, the study location, study participants, the number of participants, the NAC regimen, and outcomes. The primary outcome was all-cause mortality. Secondary outcomes included the requirement for IMV, the risk of ICU admission, the length of hospital stay, and the length of ICU stay.

### 2.4 Risk-of-bias assessment

Two investigators (PYH and THL) independently assessed the risk of bias for each included study. The revised Cochrane Risk-of-Bias tool for randomized trials (RoB-2) was used to evaluate the RCTs [23]. All RoB assessments were verified through the consensus of the entire review group. The overall certainty of the evidence was evaluated using Grades of Recommendation, Assessment, Development, and Evaluation (GRADE).

### 2.5 Statistical analysis

Statistical analysis was conducted using Review Manager (version 5.4; Nordic Cochrane Center, Copenhagen, Denmark). We calculated the risk ratio (RR) and mean difference (MD) with 95 % confidence intervals (CIs) for categorial and continuous variables, respectively. Random-effects meta-analyses were implemented to pool the data. This study used Cochran's Q test and the I^2^ statistic to assess heterogeneity. Substantial heterogeneity was indicated at I^2^ > 50 % or *p* < 0.10. Leave-one-out sensitivity analyses were employed for the primary outcome to evaluate whether individual studies significantly influenced the magnitude of the determined association between the treatment and study outcomes. Subgroup analyses by study design, the method of NAC administration, and the severity of COVID-19 were conducted.

## 3 Results

### 3.1 Search results

**Fig. 1:**
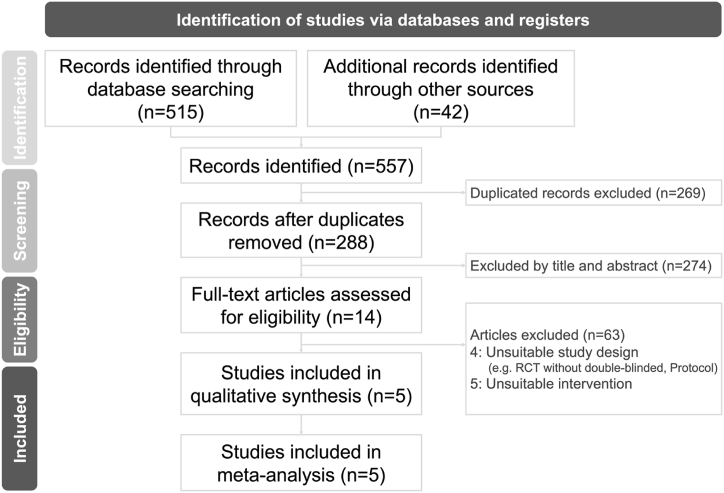
Flowchart of study selection process.

The researchers initially retrieved 557 records. After excluding 269 duplicate articles, the researchers screened 288 articles and excluded an additional 274 based on their titles and abstracts. The 14 remaining articles underwent a full-text review for eligibility assessment. Finally, this study identified a total of five RCTs [[17], [18], [19], [20], [21]] that met the inclusion criteria. The algorithm for study selection is presented in Fig. 1.

### 3.2 Characteristics of included studies

Table 1 summarizes the characteristics of the five included studies [[17], [18], [19], [20], [21]]. One study [21] was a phase 2 RCT, and the other four were phase 3 studies [[17], [18], [19], [20]]. All were single-center studies conducted in different countries, including Iran (n = 3), Brazil (n = 1), and Croatia (n = 1). With the exception of the study by Panahi et al. that did not clearly define disease severity [20], the other four studies included patients with severe COVID-19 [[17], [18], [19],^21^]. The regimen and administration of NAC differed in each RCT, including intravenous injection (n = 3) and inhalation (n = 2). Two and three RCTs used the SOC and a placebo as comparators, respectively. This study included a total of 651 patients, among which 320 received NAC as an intervention and 331 belonged to the control group, receiving either a placebo or the SOC.

**Table 1:** Characteristics of included studies.

Study	Study design	Study sites	Study period	Patients	Intervention	Comparator	No of patients	
							NAC	Control
de Alencar et al., 2021	Double-blind, randomized, placebo-controlled trial	Single center in Brazil	from April 10, 2020 to 25 May 2020	Adult patients with severe COVID-19	IV NAC with 14 g in the first 4 h and 7 g in the next 16 h	Placebo	67	68
Deli'c et al., 2022	randomized controlled trial	Single center in Croatia	Between October 2020 and June 2021	Adult patients with COVID-19 treated with mechanical ventilation	Inhaled NAC every 12 h	SOC	39	52
Mousapour et al., 2022	Double-blind, randomized, placebo-controlled trial	Single center in Iran	NA	Adult patients with severe COVID-19	IV NAC with 1 g every 12 h	Placebo	42	41
Panahi et al., 2022	Prospective, randomized, open-labeled, controlled clinical trial	Single center in Iran	from May 2021 until August 2021	Adult patients with COVID-19	NAC spray one puff (200 mg per puff) every 12 h for seven days	SOC	125	125
Taher et al., 2021	phase 2, randomized, double-blind, clinical trial	Single center in Iran	from June 2020 until February 2021	Adult COVID-19 patients with ARDS	IV NAC at a dose of 40 mg/kg/day continuous infusion for three days	Placebo	47	45

NAC, N-acetylcysteine; IV, intravenous; NA, not applicable; ARDS, acute respiratory distress syndrome; SOC, standard of care.

**Fig. 2:**
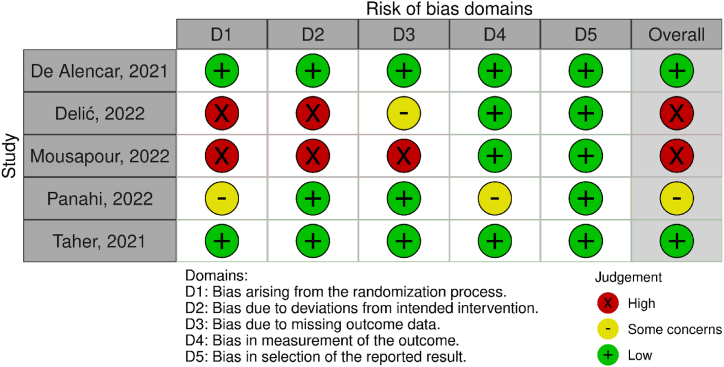
Risk-of-bias assessment.

Fig. 2 presents the quality assessment of included studies using the Cochrane RoB-2. Two studies had a low risk of bias in all domains [17,21], one study had some risk of overall bias [20], and two studies had a high risk of overall bias [18,19]. A low risk of bias was present in the selection of the reported results across all studies. A high risk of bias was primarily associated with the randomization process and deviations from the intended intervention in all the domains evaluated for the five studies. Based on the GRADE (Table 3) framework, the primary outcome was judged to be very low certainty of evidence.

**Table 2:** Findings of subgroup analyses with respect to mortality.

Subgroup	No of study	No of patients	Risk ratio	95 % confidence interval	I^2^
NAC administration					
Intravenous injection	3	310	0.90	0.55–1.46	0 %
Inhalation	2	341	0.28	0.01–5.43	97 %
Study design					
Phase 2	1	92	0.82	0.43–1.58	
Phase 3	4	559	0.53	0.15–1.85	90 %
Comparator					
Standard of care	2	341	0.28	0.01–5.43	97 %
Placebo	3	310	0.90	0.55–1.46	0 %
Severe COVID-19	4	401	0.90	0.67–1.21	0 %

**Table 3:** GRADE assessment.

Participants	Risk of bias	Inconsistency	Indirectness	Imprecision	Publication bias	Overall certainty of evidence
Mortality						
5 RCTs 651 patients	Very serious	Serious	Not serious	Very serious	Not serious	Very Low
Invasive mechanical ventilation						
3 RCTs 310 patients	Serious	Not serious	Not serious	Very serious	Not serious	Very Low
Length of hospitalization						
3 RCTs 477 patients	Very serious	Not serious	Not serious	Very serious	Not serious	Very Low
Intensive care unit stay						
2 RCTs 227 patients	Very serious	Not serious	Not serious	Very serious	Not serious	Very Low

### 3.3 Primary outcome

**Fig. 3:**
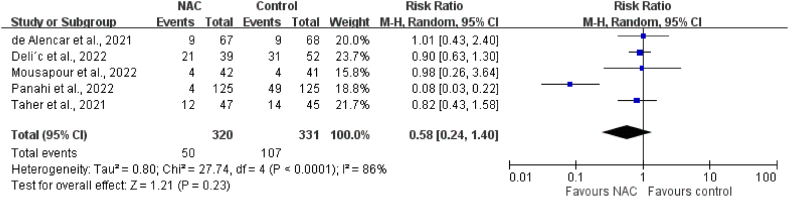
Meta-analysis of the association between N-acetylcysteine (NAC) and mortality.

The pooled analysis of five RCTs [[17], [18], [19], [20], [21]] revealed that the NAC group had a lower mortality rate compared with the control group (15.6 % [50/320] vs. 32.3 % [107/331]). However, this difference did not attain statistical significance (RR: 0.58; 95 % CI: 0.24–1.40, *p* = 0.23), and the result indicated a high level of heterogeneity (I^2^ = 86 %; *p* < 0.0001; Fig. 3). A leave-one-sensitivity test similarly detected no significant difference in mortality rate between the study and the control groups. In addition, the high heterogeneity level disappeared when excluding the study of Panahi et al. [20]. Subgroup analyses were conducted according to the study design, the method of NAC administration, the comparator type, and disease severity. None of the subgroup analysis results indicated a significant difference in mortality between the NAC and control groups (Table 2).

## 4 Secondary outcomes

**Fig. 4:**
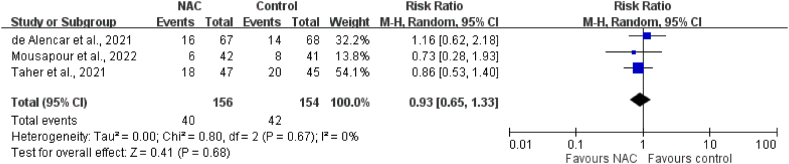
Meta-analysis of the association between N-acetylcysteine (NAC) and invasive mechanical ventilation (IMV).

**Fig. 5:**
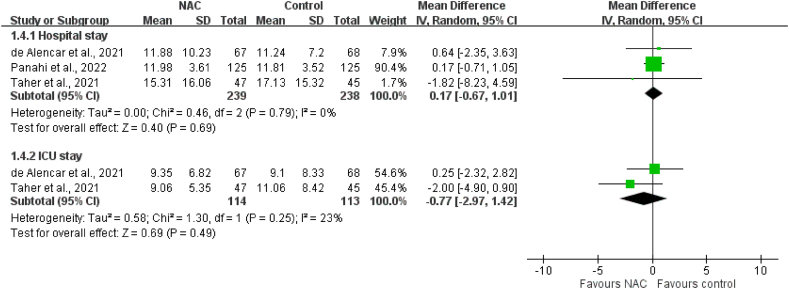
Meta-analysis of the association between N-acetylcysteine (NAC) and the length of hospitalization and intensive care unit (ICU) stay.

The pooled analysis results indicated no significant difference with respect to IMV incidence between the NAC group and the control group in three RCTs with 310 patients (RR: 0.93; 95 % CI: 0.65–1.33; *p* = 0.68); furthermore, no heterogeneity was detected (I^2^ = 0 %; *p* = 0.67; Fig. 4). Similarly, the results indicated no significant difference with respect to the risk of ICU admission between groups in the pooled analysis of two RCTs involving 385 patients (RR: 0.86; 95 % CI: 0.62–1.21; *p* = 0.39); no heterogeneity was detected (I^2^ = 0 %; *p* = 0.39). Furthermore, the results did not reveal a significant difference between groups with respect to the length of hospital stay (MD: 0.17 days; 95 % CI: −0.67–1.01; *p* = 0.69) or the length of ICU stay (MD: −0.77 days; 95 % CI: −2.97–1.42; *p* = 0.49); studies for these findings had low heterogeneity (Fig. 5).

## 5 Discussion

This meta-analysis of five RCTs [[17], [18], [19], [20], [21]] including 651 patients assessed the clinical efficacy of NAC in the treatment of patients with COVID-19. The results demonstrate that NAC did not improve the outcomes of patients with COVID-19. The following evidence supports this conclusion. First, although patients in the NAC group had a lower mortality rate compared with those in the control group, this difference was not statistically significant. Second, the results of the leave-one-out sensitivity test and the subgroup analyses by study design, NAC use, and the comparator type indicated a similar mortality rate between groups. Third, in addition to mortality, the data indicated no significant difference between the NAC group and the control groups with respect to the requirement for IMV, the incidence of ICU admission, or the length of hospitalization or ICU stay. Therefore, the study findings did not support the use of NACin the treatment of patients with COVID-19.

Our findings did not agree with those of previous cohort studies [[14], [15], [16]]. Although studies have demonstrated the benefit of NAC in patients with COVID-19 with respect to clinical recovery and inflammatory response, their findings may be limited by small sample sizes and significant selection bias [[14], [15], [16]]. Unlike our study, the RCT by Panahi et al. noted a significantly lower mortality rate in the intervention group receiving NAC than in the control group (3.2 % vs. 39.2 %, respectively; *p* < 0.001), but did not observe a difference in the length of hospital stay (11.98 ± 3.61 vs. 11.81 ± 3.52, respectively; *p* = 0.814) or in the incidence of ICU admission (7.2 % vs. 11.2 %, respectively; *p* = 0.274) [20]. However, similar to those of four RCTs [[17], [18], [19],^21^], our results indicated no survival benefit in the intervention group. In addition, our findings were based on more robust evidence compared with relevant cohort studies [[14], [15], [16]], and we included more patients than the RCT of Panahi et al. [20]. Therefore, the results did not demonstrate that NAC improved the mortality of patients with COVID-19; however, NAC may help ameliorate COVID-19-associated inflammation.

In addition to clinical efficacy, we also concerned the safety of NAC in patients with COVID-19. The study by de Alencar et al. observed no adverse events in 67 patients who received NAC; all patients tolerated the drug and the fluid dosing well [17]. Delić et al. reported that one patient (2.4 %) had bronchospasm during NAC inhalation [18]. Taher et al. noted no intolerable or severe adverse events caused by NAC infusion, and no patients dropped out of their study due to intolerable adverse events during NAC administration [21]. These findings indicate the excellent safety profile of NAC in the treatment of patients with COVID-19.

This study has several limitations. First, we included a small sample of RCTs and patients. Second, the results indicated a high level of heterogeneity in the overall analysis of mortality, possibly caused by including the Panali et al. study [20]. After excluding that study, we did not detect heterogeneity in the analysis of the four remaining RCTs [[17], [18], [19],^21^], and the similar mortality rates between groups remained unchanged. Third, variation in regimens and the administration of NAC in the included studies may result in heterogeneity. However, the findings were primarily based on no-to-low heterogeneity levels. Forth, the majority of the RCTs were conducted in Iran, which may limit this study's generalizability.

In conclusion, the present meta-analysis demonstrates that the administration of NAC did not improve the clinical outcomes of patients with COVID-19, and the researchers do not recommend its routine use for patients with SARS-CoV-2 infections.

## Ethics approval and consent to participate

This article does not contain any studies with human participants or animals performed by any of the authors.

## Consent for publication

Not applicable.

## Data availability statement

Data included in article/supp. material/referenced in article.

## Funding

None.

## CRediT authorship contribution statement

**Ting-Hui Liu:** Writing – original draft, Investigation, Formal analysis, Data curation, Conceptualization. **Jheng-Yan Wu:** Investigation, Formal analysis, Data curation. **Po-Yu Huang:** Investigation, Formal analysis, Data curation. **Ya-Wen Tsai:** Methodology, Investigation, Formal analysis, Data curation. **Wan-Hsuan Hsu:** Methodology, Investigation, Formal analysis, Data curation. **Min-Hsiang Chuang:** Methodology, Investigation, Formal analysis, Data curation. **Hung-Jen Tang:** Writing – review & editing, Supervision. **Chih-Cheng Lai:** Writing – review & editing, Writing – original draft, Supervision, Formal analysis, Data curation, Conceptualization.

## Declaration of competing interest

The authors declare that they have no known competing financial interests or personal relationships that could have appeared to influence the work reported in this paper.
