# Supplementary material for: Clinical efficacy of N-acetylcysteine for COVID-19: A systematic review and meta-analysis of randomized controlled trials
Source: Heliyon. 2024 Jan 26;10(3):e25179. doi: 10.1016/j.heliyon.2024.e25179 (PMC10839595; doi:10.1016/j.heliyon.2024.e25179)
Supplement: Multimedia component 1 [file mmc1.docx]

**Appendix 1. Search strategy**

**Database: PubMed**

| No | Query | Results |
| --- | --- | --- |
| #1 | "covid 19"[All Fields] OR "covid 19"[MeSH Terms] OR "covid 19 vaccines"[All Fields] OR "covid 19 vaccines"[MeSH Terms] OR "covid 19 serotherapy"[All Fields] OR "covid 19 nucleic acid testing"[All Fields] OR "covid 19 nucleic acid testing"[MeSH Terms] OR "covid 19 serological testing"[All Fields] OR "covid 19 serological testing"[MeSH Terms] OR "covid 19 testing"[All Fields] OR "covid 19 testing"[MeSH Terms] OR "sars cov 2"[All Fields] OR "sars cov 2"[MeSH Terms] OR "severe acute respiratory syndrome coronavirus 2"[All Fields] OR "ncov"[All Fields] OR "2019 ncov"[All Fields] OR (("coronavirus"[MeSH Terms] OR "coronavirus"[All Fields] OR "cov"[All Fields]) AND 2019/11/01:3000/12/31[Date - Publication]) | 321,917 |
| #2 | "acetylcysteine"[MeSH Terms] OR "acetylcysteine"[All Fields] OR "n acetylcysteine"[All Fields] | 21, 020 |
| #3 | ("covid 19"[All Fields] OR "covid 19"[MeSH Terms] OR "covid 19 vaccines"[All Fields] OR "covid 19 vaccines"[MeSH Terms] OR "covid 19 serotherapy"[All Fields] OR "covid 19 nucleic acid testing"[All Fields] OR "covid 19 nucleic acid testing"[MeSH Terms] OR "covid 19 serological testing"[All Fields] OR "covid 19 serological testing"[MeSH Terms] OR "covid 19 testing"[All Fields] OR "covid 19 testing"[MeSH Terms] OR "sars cov 2"[All Fields] OR "sars cov 2"[MeSH Terms] OR "severe acute respiratory syndrome coronavirus 2"[All Fields] OR "ncov"[All Fields] OR "2019 ncov"[All Fields] OR (("coronavirus"[MeSH Terms] OR "coronavirus"[All Fields] OR "cov"[All Fields]) AND 2019/11/01:3000/12/31[Date - Publication])) AND ("acetylcysteine"[MeSH Terms] OR "acetylcysteine"[All Fields] OR "n acetylcysteine"[All Fields]) | 106 |

**Database: Cochrane**

| No | Query | Results |
| --- | --- | --- |
| #1 | ("covid 19" OR "SARS-CoV-2" OR "sars-cov-2" OR "Severe Acute Respiratory Syndrome Coronavirus 2"):ti,ab,kw | 13, 683 |
| #2 | MeSH descriptor: [COVID-19] explode all trees | 2, 553 |
| #3 | "acetylcysteine" OR "acetylcysteine" OR "n acetylcysteine" | 2, 558 |
| #4 | MeSH descriptor: [Acetylcysteine] explode all trees | 1, 229 |
| #5 | (#1 OR #2) AND (#3 OR #4) | 42 |

**Database: Embase**

| No | Query | Results |
| --- | --- | --- |
| #1 | ('coronavirus disease 2019'/exp OR '2019 novel coronavirus disease' OR '2019 novel coronavirus epidemic' OR '2019 novel coronavirus infection' OR '2019-ncov disease' OR '2019-ncov infection' OR 'covid' OR 'covid 19' OR 'covid 19 induced pneumonia' OR 'covid 2019' OR 'covid-10' OR 'covid-19' OR 'covid-19 induced pneumonia' OR 'covid-19 pneumonia' OR 'covid19' OR 'sars coronavirus 2 infection' OR 'sars coronavirus 2 pneumonia' OR 'sars-cov-2 disease' OR 'sars-cov-2 infection' OR 'sars-cov-2 pneumonia' OR 'sars-cov2 disease' OR 'sars-cov2 infection' OR 'sarscov2 disease' OR 'sarscov2 infection' OR 'wuhan coronavirus disease' OR 'wuhan coronavirus infection' OR 'coronavirus disease 2' OR 'coronavirus disease 2010' OR 'coronavirus disease 2019' OR 'coronavirus disease 2019 pneumonia' OR 'coronavirus disease-19' OR 'coronavirus infection 2019' OR 'ncov 2019 disease' OR 'ncov 2019 infection' OR 'new coronavirus pneumonia' OR 'novel coronavirus 2019 disease' OR 'novel coronavirus 2019 infection' OR 'novel coronavirus disease 2019' OR 'novel coronavirus infected pneumonia' OR 'novel coronavirus infection 2019' OR 'novel coronavirus pneumonia' OR 'paucisymptomatic coronavirus disease 2019' OR 'severe acute respiratory syndrome 2' OR 'severe acute respiratory syndrome 2 pneumonia' OR 'severe acute respiratory syndrome cov-2 infection' OR 'severe acute respiratory syndrome coronavirus 2 infection' OR 'severe acute respiratory syndrome coronavirus 2019 infection') | 352, 977 |
| #2 | ('acetylcysteine'/exp OR 'acetain' OR 'acetyl cysteine' OR 'acetyl l cysteine' OR 'acetylcystein' OR 'acetylcysteine' OR 'fluimicil' OR 'fluimucil' OR 'fluimucil a' OR 'fluimucil antidote' OR 'fluimucil expectorant' OR 'fluimucil forte' OR 'n acetyl cystein' OR 'n acetyl cysteine' OR 'n acetyl l cysteine' OR 'n acetylcystein' OR 'n acetylcysteine') | 46, 193 |
| #3 | #1 AND #2 | 409 |
| #4 | #3 AND [embase]/lim NOT ([embase]/lim AND [medline]/lim) | 182 |
